# Scaling Up Single-Cell Proteomics

**DOI:** 10.1016/j.mcpro.2021.100179

**Published:** 2021-11-20

**Authors:** Nikolai Slavov

**Affiliations:** Department of Bioengineering, Northeastern University, Boston, Massachusetts, USA; Barnett Institute, Northeastern University, Boston, Massachusetts, USA

**Keywords:** single-cell biology, single-cell proteomics, single-cell proteogenomic, high-throughput proteomics, robust protocols, quality controls, data pipelines, sample preparation, multiplexed mass-spectrometry, ultrasensitive proteomics, autoPOTS, automated preparation in one pot for trace samples, DIA, data-independent acquisition, mPOP, minimal ProteOmic sample Preparation, nPOP, nano-ProteOmic sample Preparation, SCoPE-MS, Single Cell ProtEomics by MS, scRNA-Seq, single-cell RNA-Seq

## Abstract

Single-cell tandem MS has enabled analyzing hundreds of single cells per day and quantifying thousands of proteins across the cells. The broad dissemination of these capabilities can empower the dissection of pathophysiological mechanisms in heterogeneous tissues. Key requirements for achieving this goal include robust protocols performed on widely accessible hardware, robust quality controls, community standards, and automated data analysis pipelines that can pinpoint analytical problems and facilitate their timely resolution. Toward meeting these requirements, this *perspective* outlines both existing resources and outstanding opportunities, such as parallelization, for catalyzing the wide dissemination of quantitative single-cell proteomics analysis that can be scaled up to tens of thousands of single cells. Indeed, simultaneous parallelization of the analysis of peptides and single cells is a promising approach for multiplicative increase in the speed of performing deep and quantitative single-cell proteomics. The community is ready to begin a virtuous cycle of increased adoption fueling the development of more technology and resources for single-cell proteomics that in turn drive broader adoption, scientific discoveries, and clinical applications.

## Highlights

• Wide adoption of single-cell proteomics can empower biology.
• Protocols for single-cell proteomics by MS are ready for broad adoption.
• Parallelizing the analysis offers multiplicative throughput gains.
• Quantity controls and community standards are essential.
• Single-cell proteomics offers unique perspective on biology.

## Graphical Abstract

**Figure:**
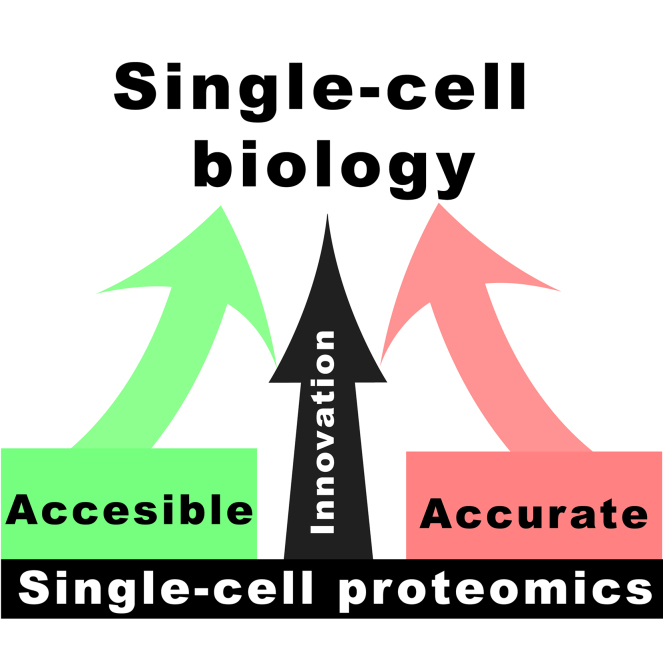

## In Brief

Single-cell proteomics will drive the next wave of single-cell biology. This requires broad adoption of existing methods, the application of rigorous quality control standards, and the continuous advancement of the technology. The advancement will be driven by numerous innovations, including highly parallelized analysis, and will increase the throughput, quantitative accuracy, and the accessibility of the single-cell proteomics.

Single-cell MS analysis of proteins has made rapid gains over the last few years (1, 2). This growth will continue since major opportunities for future technological and methodological advancements ensure that innovations will continue to drive analytical capabilities (3). Indeed, single-cell MS detects peptide ions with high sensitivity, but the proteome coverage of current methods is limited by time constraints (4). The relaxation of these constraints by innovations in data acquisition and interpretation may increase proteome coverage by 10-fold (4). At the same time, existing single-cell proteomics MS methods are reaching a maturity level that should allow their broader adoption. This *perspective* focuses on key steps needed to achieve broader adoption of single-cell proteomics by tandem MS and to scale up its throughput to tens of thousands of single cells analyzed at affordable cost and time.

## Biological Systems and Questions Demanding Single-Cell Proteomics

Single-cell analysis is trendy, but it is not always essential. It may not be essential for model systems consisting of a mostly homogeneous cellular population or consisting of well-defined discrete subpopulations, which can be isolated based on reliable markers. However, such model systems are rare, especially when working with multicellular organisms and *in vivo* samples. Indeed, even isogenic cell populations may exhibit significant biological heterogeneity (5, 6, 7). If a cellular system is assumed to be homogeneous and analyzed by bulk methods, the resulting data cannot reject the assumption of homogeneity even when it is incorrect and misleading (1). For these reasons, single-cell analysis is increasingly the method of choice, especially when working with complex biological tissues (5, 8, 9, 10). For decades, protein analysis of single mammalian cells has been performed using affinity reagents (5) while the power of MS to achieve deep proteome analysis has been limited to quantifying the average protein levels in samples consisting of many (often heterogeneous) cells (11, 12, 13, 14). However, increasingly, MS laboratories are succeeding in bringing the power of MS analysis to quantitative protein analysis of single mammalian cells (2, 15).

Single-cell proteomics is rapidly developing in the wake of single-cell RNA-Seq (scRNA-Seq), which prompts the question of when to use scRNA-Seq and when to use single-cell proteomics. The simple answer is to measure RNAs if interested in RNAs and measure proteins if interested in proteins. This simple answer is complicated by hopes that mRNA levels are reliable surrogates for protein levels (16). The degree to which mRNA levels may be used as surrogates for protein levels has received considerable attention and borne out controversy. The controversy stems in part from studies not accounting for measurement error. Yet, measurement errors may contribute significantly to the measured difference between RNA and protein abundances, and this contribution must be explicitly accounted for (17, 18). These errors stem from technical variability in sample collection, preparation, and measurement and can be empirically estimated from independent measurements (18). After accounting for differences because of measurement noise, mRNA levels remain poor substitutes for the levels of proteins and proteoforms because much of the protein abundance variation across human tissues likely stems from post-transcriptional regulation (18). The role of post-transcriptional regulation is particularly strong for some proteins, such as those forming complexes, and generally can extend to the entire proteome in a condition-specific manner (16, 19).

Thus, instead of assuming that RNA levels faithfully reflect protein levels, we should measure both proteins and RNAs. Such joint measurements can reveal regulatory mechanisms. For example, covariation between the levels of transcription factors and mRNAs may suggest transcriptional regulation, whereas divergence between the RNA and protein levels of a gene may suggest post-transcriptional regulation of protein synthesis or degradation. Thus, joint single-cell proteogenomic analysis may enable characterizing both transcriptional and post-transcriptional regulation in single cells (19, 20, 21). Indeed, combined analysis of single-cell transcriptomics and proteomics data can detect covariation between transcription factors, such as p53, and their target transcripts, thus revealing transcriptional regulation not detectable from single-cell RNA data alone (21). Such examples are early harbingers for the potential of single-cell proteomics to identify mechanisms of biological regulation in health and disease (10).

## Trade-Offs Between Single-Cell Proteomics Methods

This increased appreciation of the need to perform single-cell protein measurements has stimulated the development of single-cell MS methods that can identify and quantify hundreds of proteins from single cells at an unprecedented scale (21, 22, 23, 24, 25, 26, 27, 28, 29). These methods aim to achieve similar objectives, such as efficient delivery of peptides from single cells to the MS instruments *via* miniaturized sample preparation (1), but differ in the approaches used for achieving these objectives. For example, sample preparation volumes can be reduced by using microfabricated wells (30) or by using droplets on the surface of a slide (31). All single-cell MS methods can be classified either as label free or as multiplexed, and these categories have associated advantages and disadvantages as previously reviewed (1, 2). An advantage of multiplexed methods for single-cell proteomics is that they can afford analyzing more cells per unit time. Since this increased throughput is relevant to scaling up the analysis to thousands of single cells, the rest of this *perspective* will focus on multiplexed methods albeit much of the discussion will be relevant to label-free methods as well.

Methods for multiplexed single-cell proteomics have relied primarily on using isobaric mass tags, usually combined with the isobaric carrier approach (32). This approach was introduced by Single-Cell ProtEomics by MS (SCoPE-MS) (22) and has been incorporated in its second version SCoPE2 (21) and other highly similar methods (25, 26, 33, 34). This approach has also allowed deep proteome quantification from small cancer samples (35) and increased sensitivity of thermal proteome profiling (36). Using the TMTpro 18-plex reagents (37), multiplexed single-cell proteomics methods can quantify thousands of proteins across thousands of individual cells within weeks and thus generate single-cell data at a comparable scale to multiwell-based scRNA-Seq methods (38). A major difference from comparable scRNA-Seq methods is that multiplexed single-cell proteomics methods have not yet become as widely employed. Thus, achieving wide adoption represents an opportunity to advance single-cell biology and biomedical research more generally.

Two complementary requirements to scaling up single-cell proteomics are (i) making the approaches robust and widely available, that is, accessibility and (ii) increasing the number of cells that can be analyzed per project, that is, throughput. These requirements are discussed below, both their state and their prospects for further development.

## Increasing Robustness and Accessibility

**Fig. 1:**
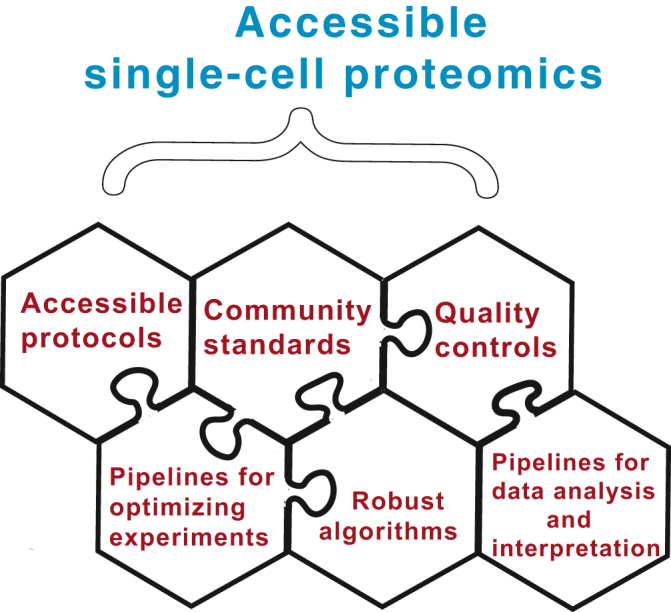
**Factors required for making single-cell proteomics broadly accessible.** The wide adoption of single-cell proteomics requires a number of factors interacting synergistically as displayed with the interlocking puzzle pieces.

Ideally, any laboratory capable of performing quantitative MS proteomics should be able to perform quantitative protein analysis in single cells. Achieving this goal requires robust single-cell proteomics protocols that can be performed on widely available equipment (Fig. 1). This requirement is sometimes incompatible with achieving the highest performance since the highest performance may require custom solutions that are challenging to implement, such as very low flow rate chromatographic separation on home-packed columns. Such high-performance solutions play a major role in driving technological developments and should be pursued in parallel with protocols aiming for robustness and accessibility.

The aim for robustness and accessibility has been a guiding principle in the development of the SCoPE2 protocol (39). Specifically, the protocol uses only commercially available equipment that is available to most core facilities and MS laboratories. Similarly, other protocols such as automated multiwell plate sample preparation (40, 41) are amenable to wide adoption. Such protocols can already be implemented by the MS community and thus can support the first wave of broader adoption of single-cell proteomics. It is imperative that accessible protocols are applied with essential controls: In the absence of controls, failures because of incorrect implementation of the protocols may be misattributed to poor performance to the methods (39). Such failures and misattribution can setback the progress of single-cell proteomics.

A major impediment to implementing MS methods can be the overhead associated with method optimization for each project, which may be very time consuming. This overhead may be reduced by highly detailed protocols that minimize the rediscovery of pitfalls. Nonetheless, even the best protocols tend to require some adaptation and troubleshooting. These aspects can be greatly facilitated by user-friendly computational pipelines that allow for quick diagnosis of problems and parameter optimization (Fig. 1). Examples of such pipelines developed for single-cell proteomics include data-driven optimization of MS (42) and the SCPcompanion (43). Such pipelines can reduce the overhead associated with method adaptation and pinpoint analytic parameters that need adjustment. For example, data-driven optimization of MS automatically evaluates factors required for quantitative single-cell proteomics by SCoPE2, such as high labeling efficiency and sampling close to the apices of elution peaks. Such computational pipelines are likely to facilitate the broader adoption of single-cell proteomics methods and their adaptation to different sample types and priorities, such as setting the desired balance between number of analyzed proteins and number of sampled protein copies per cell (32). Tools that are not specific for single-cell proteomics (as reviewed in Ref. (44)) can also provide useful functionality, such as data exploration and visualization (45, 46). The development and further refinement of easy-to-use pipelines for optimizing data acquisition and evaluating sample and data quality is an important investment toward significantly reducing the overhead of adopting single-cell proteomics methods.

Making single-cell proteomics accessible also demands accessible computational pipelines for data analysis and interpretation. Currently, several pipelines are available for data processing, including the SCoPE2 pipeline (https://doi.org/10.5281/zenodo.4339954), its implementation in the scp Bioconductor package that offers increased functionality (47), and SCeptre (25). The SCoPE2 pipeline and the scp package are implemented in the R programming language, whereas SCeptre is implemented in Python. These pipelines can provide the initial data processing from search engine output to data matrices, which then can be analyzed further by computational tools developed for scRNA-Seq data, as in the case of joint projection of mRNA and protein data with Conos (21, 48). Thus, the existing software packages already provide a functional toolset that is certain to grow in a positive feedback loop with the increased adoption of single-cell proteomics across the community. This growth should include error estimation and propagation algorithms informed by the characteristics of the measurement noise in single-cell MS data. Furthermore, we should expand the pipeline functionality that quantifies the dependence of the final results on the choice of data processing steps. For example, reporting whether the identification of a subpopulation of single cells depends on the method used for batch correction.

All data processing pipelines should transparently report quality control metrics based on consensus community standards (Fig. 1). Such community standards are urgently needed to support the wider adoption and scaling up of single-cell proteomics. Specifically, these metrics must distinguish between reproducibility and quantitative accuracy, between accuracy of relative and absolute quantification, between the variety of approaches used for computing coefficients of variation, and many other quantitative measurements that are currently conflated in single-cell MS publications (18, 49, 50). These community standards should reflect a broad consensus, and indeed conference workshops have begun discussions toward formulating such standards (http://workshop2019.single-cell.net/). This important next step should be established by an authoritative white paper articulating best practices and recommending quantitative benchmarks and data reporting formats.

## Increasing the Throughput of Single-Cell Proteomics

High throughput is essential for many biological investigations, especially for achieving high enough statistical power (51). In the case of single-cell analysis, throughput is also essential to enable the analysis of a large enough number of single cells to have a chance to sample rare cells (8, 10). The throughput of single-cell proteomics is determined both by the throughput of sample preparation and by the throughput of MS analysis.

## Highly Parallel Sample Preparation

Just a few years ago, relatively few single cells could be simultaneously prepared for analysis (30, 52, 53), and thus, sample preparation was a limiting step. Sample preparation throughput increased with the introduction of automated multiwell-plate methods, such as minimal ProteOmic sample Preparation (mPOP) (21, 39, 40) and automated preparation in one pot for trace samples (autoPOTS) (41). A further increase is afforded by a droplet sample preparation method (nano-ProteOmic sample Preparation [nPOP]) that enables the simultaneous and automated preparation of over 2000 single cells in droplets on a slide surface (31). In addition to increasing throughput, the simultaneous processing of thousands of cells reduces the batch effects associated with different sample preparation batches.

While nPOP uses only commercially available equipment and reagents, the equipment is expensive and not widely available (31). Thus, nPOP is less accessible than mPOP and autoPOTS. This example illustrates the tradeoff between high performance, in this case, simultaneous preparation of thousands of single cells in 20 nl reaction volumes, and the most accessible protocols, mPOP and autoPOTS. Importantly, the accessible protocols can support high-quality sample preparation and can empower single-cell proteomics analysis even for laboratories that do not have access to expensive equipment.

## Parallel Analysis of Both Peptides and Single Cells

**Fig. 2:**
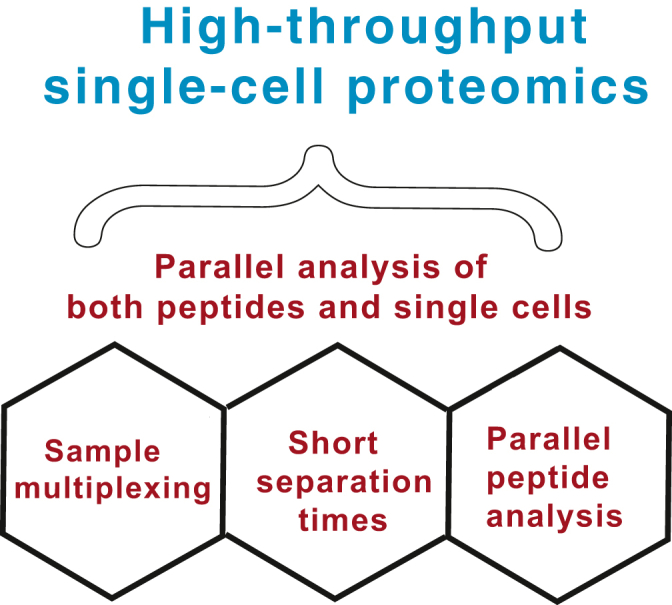
**Strategies for increasing the throughput of single-cell proteomics.** The throughput can be increased by (i) parallel analysis of single cells *via* sample multiplexing, parallel analysis of peptides *via* DIA, and (iii) shorter separation times, which reduce the MS time per labeled set. Combining the gains from these three approaches can multiplicatively increase the throughput of single-cell proteomics (54). DIA, data-independent acquisition.

As the rate of robust sample preparation has increased, the rate of MS analysis of samples has become limiting. The two principal approaches to relieving this limitation are (i) increased multiplexing and (ii) decreased MS time per sample (Fig. 2). Increased multiplexing is particularly attractive as it may be combined with pooling peptide fragments across single cells and thus enhance peptide sequence identification (32). Furthermore, the relatively small protein amount per single cell implies that increased multiplexing should not limit the copy number of ions sampled per single cell (1). These advantages will likely motivate the development of higher plex reagents for single-cell proteomics. While such development requires significant investments for isobaric mass tags (37), nonisobaric isotopologous mass tags may be easier to develop and may enable both high sensitivity and high throughput (54).

Multiplexing single-cell proteomics can introduce batch effects and help mitigate them. Variability between batches of mass tags or tag-specific biases could result in batch effects. Such problems can be minimized by using high-quality isotopologous mass tags. Batch effects originating from multiplexing can be reduced by using reference samples to normalize for set-specific artifacts (21, 39). Experimental strategies that minimize set-specific biases can further minimize batch effects. For example, nonisobaric multiplexing avoids biases because of coisolating isobarically labeled peptides (54). While such experimental design strategies can reduce batch effects, some batch effects will remain and may require computational corrections.

The second approach to increased MS throughput is decreased MS time per sample (Fig. 2). Such decrease will reduce the number of peptides that can be analyzed by data-dependent acquisition but may support high-throughput analysis by data-independent acquisition (DIA) (4, 54), as demonstrated with bulk samples (51, 55). The high flow rates used with ultra-fast bulk DIA analysis are incompatible with maximizing MS sensitivity, but nonetheless shorter gradients may speed up single-cell analysis as well. Indeed, this possibility has been demonstrated with label-free DIA analysis of single HeLa cells utilizing 30 min of chromatographic gradients (29). Yet, much shorter gradients are required if label-free analysis is to match the throughput of multiplexed methods.

Ideally, throughput can be synergistically increased by combining short separation times and parallel analysis of both peptides and single cells (Fig. 2). This combination may be achieved by multiplexed DIA performed on short gradients: It multiplies the advantages of sample multiplexing, parallel peptide analysis, and short MS analysis time per sample (1, 4, 54). Multiplexing DIA with 3-plex nonisobaric isotopologous mass tags allows for threefold increased throughput without reduction of proteome coverage or quantitative accuracy (54). This strategy, termed plexDIA, allows for accurate protein quantification at both MS1 and MS2 levels (54). It was enabled by advances in data interpretation and can be further advanced by improving data interpretation, such as peptide sequence propagation within a labeled set. Another major opportunity for advancing plexDIA is the development of higher plex nonisobaric mass tags. Indeed, if scaled to higher plex, plexDIA can provide a substantial increase in the sensitivity, quantitative accuracy, and throughput of single-cell proteomics (4, 54). Combination of increased DIA multiplexing and short separation gradients appears the most promising strategy for achieving high-throughput and high-depth quantitative single-cell proteomics.

## Conclusion

Increasing appreciation for the need to perform single-cell protein analysis has propelled the field of single-cell proteomics by MS, resulting in methods that allow quantifying over a thousand proteins per cell while analyzing hundreds of single cells per day. Taking advantage of these capabilities requires their broad dissemination, which in turn requires robust and accessible protocols and data analysis pipelines. These requirements are already available to support the first wave of technology dissemination, and this dissemination will in turn drive the development of more analytical and computational tools. Central to the success of this virtuous cycle is a set of community standards that will ensure rigor in data reporting and interpretation. The stage is set for scaling up single-cell proteomics to the challenges and opportunities of cutting-edge biomedical research.

## Conflict of interest

The author has filed patents for single-cell proteomics methods.

## References

[1] Slavov N. (2021). Single-cell protein analysis by mass spectrometry. Curr. Opin. Chem. Biol..

[2] Clark N.M., Elmore J.M., Walley J.W. (2021). To the proteome and beyond: Advances in single-cell omics profiling for plant systems. Plant Physiol..

[3] Specht H., Slavov N. (2018). Transformative opportunities for single-cell proteomics. J. Proteome Res..

[4] Slavov N. (2021). Driving single cell proteomics forward with innovation. J. Proteome Res..

[5] Levy E., Slavov N. (2018). Single cell protein analysis for systems biology. Essays Biochem..

[6] Eldar A., Elowitz M.B. (2010). Functional roles for noise in genetic circuits. Nature.

[7] Raj A., van Oudenaarden A. (2008). Nature, nurture, or chance: Stochastic gene expression and its consequences. Cell.

[8] Tanay A., Regev A. (2017). Scaling single-cell genomics from phenomenology to mechanism. Nature.

[9] Regev A., Teichmann S.A., Lander E.S., Amit I., Benoist C., Birney E., Bodenmiller B., Campbell P., Carninci P., Clatworthy M., Others (2017). Science forum: The human cell atlas. Elife.

[10] Slavov N. (2020). Unpicking the proteome in single cells. Science.

[11] Zhang B., Wang J., Wang X., Zhu J., Liu Q., Shi Z., Chambers M.C., Zimmerman L.J., Shaddox K.F., Kim S., Davies S.R., Wang S., Wang P., Kinsinger C.R., Rivers R.C. (2014). Proteogenomic characterization of human colon and rectal cancer. Nature.

[12] Harel M., Ortenberg R., Varanasi S.K., Mangalhara K.C., Mardamshina M., Markovits E., Baruch E.N., Tripple V., Arama-Chayoth M., Greenberg E., Shenoy A., Ayasun R., Knafo N., Xu S., Anafi L. (2019). Proteomics of melanoma response to immunotherapy reveals mitochondrial dependence. Cell.

[13] Bamberger C., Pankow S., Martínez-Bartolomé S., Ma M., Diedrich J., Rissman R.A., Yates J.R. (2021). Protein footprinting via covalent protein painting reveals structural changes of the proteome in Alzheimer’s disease. J. Proteome Res..

[14] Mertins P., Mani D.R., Ruggles K.V., Gillette M.A., Clauser K.R., Wang P., Wang X., Qiao J.W., Cao S., Petralia F., Kawaler E., Mundt F., Krug K., Tu Z., Lei J.T. (2016). Proteogenomics connects somatic mutations to signalling in breast cancer. Nature.

[15] Marx V. (2019). A dream of single-cell proteomics. Nat. Methods.

[16] Liu Y., Beyer A., Aebersold R. (2016). On the dependency of cellular protein levels on mRNA abundance. Cell.

[17] Csárdi G., Franks A., Choi D.S., Airoldi E.M., Drummond D.A. (2015). Accounting for experimental noise reveals that mRNA levels, amplified by post-transcriptional processes, largely determine steady-state protein levels in yeast. PLoS Genet..

[18] Franks A., Airoldi E., Slavov N. (2017). Post-transcriptional regulation across human tissues. PLoS Comput. Biol..

[19] Buccitelli C., Selbach M. (2020). mRNAs, proteins and the emerging principles of gene expression control. Nat. Rev. Genet..

[20] Mahdessian D., Cesnik A.J., Gnann C., Danielsson F., Stenström L., Arif M., Zhang C., Le T., Johansson F., Shutten R., Bäckström A., Axelsson U., Thul P., Cho N.H., Carja O. (2021). Spatiotemporal dissection of the cell cycle with single-cell proteogenomics. Nature.

[21] Specht H., Emmott E., Petelski A.A., Huffman R.G., Perlman D.H., Serra M., Kharchenko P., Koller A., Slavov N. (2021). Single-cell proteomic and transcriptomic analysis of macrophage heterogeneity using SCoPE2. Genome Biol..

[22] Budnik B., Levy E., Harmange G., Slavov N. (2018). SCoPE-MS: Mass spectrometry of single mammalian cells quantifies proteome heterogeneity during cell differentiation. Genome Biol..

[23] Cong Y., Motamedchaboki K., Misal S.A., Liang Y., Guise A.J., Truong T., Huguet R., Plowey E.D., Zhu Y., Lopez-Ferrer D., Kelly R.T. (2021). Ultrasensitive single-cell proteomics workflow identifies >1000 protein groups per mammalian cell. Chem. Sci..

[24] Lombard-Banek C., Moody S.A., Manzini M.C., Nemes P. (2019). Microsampling capillary electrophoresis mass spectrometry enables single-cell proteomics in complex tissues: Developing cell clones in live Xenopus laevis and zebrafish embryos. Anal. Chem..

[25] Schoof E.M., Furtwängler B., Üresin N., Rapin N., Savickas S., Gentil C., Lechman E., Keller U., auf D., Dick J.E., Porse B.T. (2021). Quantitative single-cell proteomics as a tool to characterize cellular hierarchies. Nat. Commun..

[26] Singh A. (2021). Towards resolving proteomes in single cells. Nat. Methods.

[27] Furtwängler B., Üresin N., Motamedchaboki K., Huguet R., Lopez-Ferrer D., Zabrouskov V., Porse B.T., Schoof E.M. (2021). Real-time search assisted acquisition on a tribrid mass spectrometer improves coverage in multiplexed single-cell proteomics. bioRxiv.

[28] Virant-Klun I., Leicht S., Hughes C., Krijgsveld J. (2016). Identification of maturation-specific proteins by single-cell proteomics of human oocytes. Mol. Cell. Proteomics.

[29] Brunner A.D., Thielert M., Vasilopoulou C.G., Ammar C. (2021). Ultra-high sensitivity mass spectrometry quantifies single-cell proteome changes upon perturbation. bioRxiv.

[30] Zhu Y., Piehowski P.D., Zhao R., Chen J., Shen Y., Moore R.J., Shukla A.K., Petyuk V.A., Campbell-Thompson M., Mathews C.E., Smith R.D., Qian W.-J., Kelly R.T. (2018). Nanodroplet processing platform for deep and quantitative proteome profiling of 10–100 mammalian cells. Nat. Commun..

[31] Leduc A., Huffman R.G., Slavov N. (2021). Droplet sample preparation for single-cell proteomics applied to the cell cycle. bioRxiv.

[32] Specht H., Slavov N. (2021). Optimizing accuracy and depth of protein quantification in experiments using isobaric carriers. J. Proteome Res..

[33] Dou M., Clair G., Tsai C.-F., Xu K., Chrisler W.B., Sontag R.L., Zhao R., Moore R.J., Liu T., Pasa-Tolic L., Smith R.D., Shi T., Adkins J.N., Qian W.-J., Kelly R.T. (2019). High-throughput single cell proteomics enabled by multiplex isobaric labeling in a nanodroplet sample preparation platform. Anal. Chem..

[34] Tsai C.-F., Zhao R., Williams S.M., Moore R.J., Schultz K., Chrisler W.B., Pasa-Tolic L., Rodland K.D., Smith R.D., Shi T., Zhu Y., Liu T. (2020). An improved boosting to amplify signal with isobaric labeling (iBASIL) strategy for precise quantitative single-cell proteomics. Mol. Cell. Proteomics.

[35] Friedrich C., Schallenberg S., Kirchner M., Ziehm M., Niquet S., Haji M., Beier C., Neudecker J., Klauschen F., Mertins P. (2021). Comprehensive micro-scaled proteome and phosphoproteome characterization of archived retrospective cancer repositories. Nat. Commun..

[36] Peck Justice S.A., McCracken N.A., Victorino J.F., Qi G.D., Wijeratne A.B., Mosley A.L. (2021). Boosting detection of low-abundance proteins in thermal proteome profiling experiments by addition of an isobaric trigger channel to TMT multiplexes. Anal. Chem..

[37] Li J., Cai Z., Bomgarden R.D., Pike I., Kuhn K., Rogers J.C., Roberts T.M., Gygi S.P., Paulo J.A. (2021). TMTpro-18plex: The expanded and complete set of TMTpro reagents for sample multiplexing. J. Proteome Res..

[38] Ziegenhain C., Vieth B., Parekh S., Reinius B., Guillaumet-Adkins A., Smets M., Leonhardt H., Heyn H., Hellmann I., Enard W. (2017). Comparative analysis of single-cell RNA sequencing methods. Mol. Cell.

[39] Petelski A.A., Emmott E., Leduc A., Gray Huffman R., Specht H., Perlman D.H., Slavov N. (2021). Multiplexed single-cell proteomics using SCoPE2. Nat. Protoc..

[40] Specht H., Harmange G., Perlman D.H., Emmott E. (2018). Automated sample preparation for high-throughput single-cell proteomics. bioRxiv.

[41] Liang Y., Acor H., McCown M.A., Nwosu A.J., Boekweg H., Axtell N.B., Truong T., Cong Y., Payne S.H., Kelly R.T. (2021). Fully automated sample processing and analysis workflow for low-input proteome profiling. Anal. Chem..

[42] Huffman R.G., Chen A., Specht H., Slavov N. (2019). DO-MS: Data-driven optimization of mass spectrometry methods. J. Proteome Res..

[43] Cheung T.K., Lee C.-Y., Bayer F.P., McCoy A., Kuster B., Rose C.M. (2021). Defining the carrier proteome limit for single-cell proteomics. Nat. Methods.

[44] Bittremieux W., Valkenborg D., Martens L., Laukens K. (2017). Computational quality control tools for mass spectrometry proteomics. Proteomics.

[45] Gatto L., Breckels L.M., Naake T., Gibb S. (2015). Visualization of proteomics data using R and bioconductor. Proteomics.

[46] Gatto L., Gibb S., Rainer J. (2020). MSnbase, efficient and elegant R-based processing and visualization of raw mass spectrometry data. J. Proteome Res..

[47] Vanderaa C., Gatto L. (2021). Replication of single-cell proteomics data reveals important computational challenges. Expert Rev. Proteomics.

[48] Barkas N., Petukhov V., Nikolaeva D., Lozinsky Y., Demharter S., Khodosevich K., Kharchenko P.V. (2019). Joint analysis of heterogeneous single-cell RNA-seq dataset collections. Nat. Methods.

[49] Peng M., Taouatas N., Cappadona S., van Breukelen B., Mohammed S., Scholten A., Heck A.J.R. (2012). Protease bias in absolute protein quantitation. Nat. Methods.

[50] Petelski A.A., Slavov N. (2020). Analyzing ribosome remodeling in health and disease. Proteomics.

[51] Slavov N. (2021). Increasing proteomics throughput. Nat. Biotechnol..

[52] Budnik B., Levy E., Slavov N. (2017). Mass-spectrometry of single mammalian cells quantifies proteome heterogeneity during cell differentiation. bioRxiv.

[53] Li Z.-Y., Huang M., Wang X.-K., Zhu Y., Li J.-S., Wong C.C.L., Fang Q. (2018). Nanoliter-scale oil-air-droplet chip-based single cell proteomic analysis. Anal. Chem..

[54] Derks J., Leduc A., Gray Huffman R., Specht H., Ralser M., Demichev V., Slavov N. (2021). Increasing the throughput of sensitive proteomics by plexDIA. bioRxiv.

[55] Messner C.B., Demichev V., Bloomfield N., Jason S.L., White M., Kreidl M., Egger A.-S., Freiwald A., Ivosev G., Wasim F., Others (2021). Ultra-fast proteomics with scanning SWATH. Nat. Biotechnol..

